# Thus spoke peptides: SARS-CoV-2 spike gene evolved in humans and then shortly in rats while the rest of its genome in horseshoe bats and then in treeshrews

**DOI:** 10.1080/19420889.2022.2057010

**Published:** 2022-04-10

**Authors:** Jaroslav Flegr, Daniel Zahradník, Michaela Zemková

**Affiliations:** a Department of Philosophy and History of Science, Faculty of Science, Charles University, Prague, Czech Republic; b Department of Forest Management, Faculty of Forestry and Wood Sciences, Czech University of Life Sciences Prague, Prague-Suchdol, Czech Republic; c Department of Biological Risks, The Silva Tarouca Research Institute for Landscape and Ornamental Gardening, Průhonice, Czech Republic

**Keywords:** SARS-CoV-2, Covid-19, host specificity, immunological distance, origin, alignment-free method, peptide vocabulary analysis

## Abstract

SARS-CoV-2 is suspected to be the product of a natural or artificial recombination of two viruses – one adapted to the horseshoe bat and the other, donor of the spike protein gene, adapted to an unknown species. Here we used a new method to search for the original host of the ancestor of the SARS-CoV-2 virus and for the donor of its gene for the spike protein, the molecule responsible for binding to and entering human cells. We computed immunological T-distances (the number of different peptides that are present in the viral proteins but absent in proteins of the host) between 11 species of coronaviruses and 38 representatives of the main mammal clades. Analyses of pentapeptides, the presumed principal targets of T-cell non-self recognition, showed the smallest T-distance of the spike protein of SARS-CoV-2 to humans, while the rest of SARS-CoV-2 proteome to the horseshoe bat. This suggests that the ancestor of SARS-CoV-2 was adapted to bats, but the spike gene donor was adapted to humans. Further analyses suggest that the ancestral coronavirus adapted to bats was shortly passaged in treeshrews, while the donor of the spike gene was shortly passaged in rats before the recombination event.

## Introduction

Coronavirus SARS-CoV-2 is the agent of Covid-19 disease and the cause of the current deadly pandemic. Covid-19 progresses to a severe acute respiratory syndrome (SARS) in 30% of hospitalized patients. Since its first outbreak in the Chinese Wuhan province in December 2019 and until January 2022, SARS-CoV-2 infected minimally 300 million and killed minimally 5.4 million people on five continents (https://ourworldindata.org).

The origin of the new coronavirus is unknown [1]. It is mostly supposed that it has been transmitted to humans from its original host, probably the horseshoe bat (genus *Rhinolophus*), via an unknown intermediate animal host. The most serious medical problem of SARS-CoV-2 is the pre-adaptation of its spike protein, the 1,273 amino acids-long product of the S gene, to infecting human cells that bear angiotensin-converting enzyme 2 (ACE2) surface proteins [2]. The affinity of SARS-CoV-2 spike protein to human ACE2 is 10–20 times higher than that of SARS-CoV-1 [3,4]. The spike protein of even the first isolates from 2019 Wuhan patients showed a perfect adaptation for entering human cells and markedly poor ability to enter the cells of bats [5]. The spike protein of SARS-CoV-2 contains a polybasic furin cleavage site not found in SARS-CoV-1 or any other B-lineage Betacoronavirus [6], including its probable recent ancestor RaTG13 [7,8]. Proteolytic cleavage of the spike protein in the furin cleavage site significantly increases the spike’s affinity to human ACE2 molecules [9], a feature that makes the furin cleavage site an important virulence factor for various kinds of viruses, including some highly pathogenic strains of artificially modified influenza [10]. In the SARS-CoV-2 – but not in any other known human coronavirus – the polybasic cleavage site evolved either convergently, by insertion of 12 nucleotides including two adjacent CGG triplets (which are present only four times in the rest of the SARS-CoV-2 genome) in an unknown intermediate animal host [7], or else it resulted from a recombination event between viruses of different genera. Such recombination of unrelated coronaviruses has not been, however, reported ever before [11].

For colonization of a new host to be successful, a virus must acquire not only the ability to infect its cells but also escape detection by or attacks of the host immune system. The process of recognition of non-self peptides (peptides present in the proteins of the parasite but not in the proteins of a host) by host’s T-cells plays a central role in both cellular and humoral immunity of vertebrates [12]. Each naïve T-cell carries a population of identical molecules of the T-cell receptor on its cell membrane with affinity to one particular peptide, or a small group of similar peptides, either non-self or self, presented as bound to major histocompatibility complex proteins (MHC proteins) on the surface of cells of the host. In the process of their maturation in the thymus, all T-cells with T-cell receptors that recognize any peptides that are contained in the host proteins and presented as bound to MHC proteins on the surface of specialized cells in a thymus are eliminated or incapacitated. A great majority of mature T-cells leaving the thymus and patrolling in the host body can therefore recognize only non-self peptides.

For both hosts and parasites, it is thus advantageous to have as narrow a peptide vocabulary as possible. The narrowest peptide vocabulary that is still compatible with sustaining all vital biological functions of proteins helps the host maintain a maximally broad repertoire of receptors on mature T-cells and thus recognize the broadest possible spectrum of peptides of parasites. For parasitic organisms, it is even more important to keep a narrow peptide vocabulary: it helps them avoid attacks of the host immune system, because parasites with a narrow peptide vocabulary carry only a small number of potential targets for T-cell recognition [13].

Indeed, the analysis of peptide vocabularies of a large set of parasitic and nonparasitic species had confirmed that the selection pressure for a narrow peptide vocabulary is stronger in parasites than in non-parasites [14]. The data show that parasitic organisms use a markedly lower number of different tetrapeptides and, especially, pentapeptides than nonparasitic organisms do. At the same time, they use a higher number of different hexapeptides in their proteins, possibly to sustain the biological functions of their proteins by compensating for the lower variety of pentapeptides.

It is highly probable that parasites, especially those with small proteomes (such as viruses) and those with a narrow host specificity, adapt to their vertebrate hosts by eliminating from their proteins those tetra- and pentapeptides that are not present in the proteins of their host. Over time, peptide vocabularies of the host and its specific parasites thus become increasingly similar, which is reflected in a number of peptides present in the proteins of the parasite but not in the proteins of the host. This is the parameter we call “immunological T-distance” [13]. The lower the T-distance of the parasite from the host (after correction for the size of proteomes and richness of host and parasite vocabularies, see Materials and Methods), the better is the parasite adapted to a particular host species. It can thus be expected that immunological T-distance between a parasite and host species is the shortest for that host species that the parasite is adapted to, in other words, the distance is smallest between a parasite and its natural host.

Here, we used an analysis of T-distances between parasites and hosts to search for the original host specificity of ancestor of the SARS-CoV-2 virus. To find the animal species the virus is best adapted to, i.e., the most likely natural host of the SARS-CoV-2 ancestor, we computed the T-distances between SARS-CoV-2 and 38 representatives of all major phylogenetic clades of mammals. To reveal the host specificity of a hypothetical donor of the spike protein gene [4,15], we computed the T-distances separately for the spike protein and the rest of the coronavirus’s proteome. As a control, we repeated the analyses with ten other animal and human coronaviruses, including RaTG13, a virus isolated in a mine colonized by bats near Tongguanzhen (Mojiang, Yunnan), which was partly sequenced in 2016 and fully sequenced in 2020 by the same team as SARS-CoV-2 [1,8].

## Methods

To compute the T-distances, we used the original sequence of the SARS-CoV-2 (GCF_009858895.2) published in January 2020 [1]. All proteomes (predicted sets of all proteins of a given organism) were downloaded from the NCBI GenBank database (see SI Tables S7–S8). When choosing the representatives of mammalian clades, we tried to avoid species that are known to be or could be interspecific hybrids, that is, those that might have proteins from two different parental species and therefore an artificially inflated peptide vocabulary. When choosing representatives of mammal clades for the analysis, we favored free-living species above domesticants, because the latter had been exposed to the same parasites as humans in the past and may have therefore partly ‘humanized’ peptidomes. Specifically, the proteins of domesticants and humans had been exposed to a long-term selection for elimination of particular peptides by a similar range of viruses [13]. It should be noted, though, that conclusions of the present study would be the same regardless of domesticants and probable interspecific hybrids being included. We used two marsupial species, the koala and the gray short-tailed opossum, as the outgroups of placental mammals, and the platypus as the outgroup of live-bearing mammals.

First, we preprocessed the proteomes of all viruses and mammals [14], that is, we filtered out all comments, annotations, and special codes (e.g., for unknown amino acids and gaps). Different species have different contents of paralogs and homologs: we have therefore kept only one representative of each protein family by eliminating all proteins that had at least one of the 16-peptides present in other already processed proteins in the proteome of a species. This helped us minimize the artificial decrease of peptide vocabulary size in paralog/homolog-rich proteomes in subsequent data-sampling. Next, we prepared lists of all unique pentapeptides and hexapeptides present in the peptidomes of all species included in the study (their pentapeptide and hexapeptide vocabularies) using the program *ImunDist 2.0 [https://figshare.com/articles/software/ImunDist/17711474]*. This program, *in silico*, cuts the proteins to overlapping peptides of a desired length (e.g., pentapeptides), records a list of unique peptides of that length (assembles a peptide vocabulary), and calculates the size of this vocabulary, that is, the number of unique peptides of a particular length in the proteome.

To calculate T-distances between the virus and potential hosts, we calculated the number of different pentapeptides (or hexapeptides) present in the peptide vocabulary of the virus (or in the spike protein of the virus/all proteins of the virus except the spike protein) but not in the peptide vocabulary of the mammalian species. To control for the effect of different sizes of mammalian proteomes (9.6–11.2 MB) and to quantify the variance in T-distance, we computed each peptide vocabulary ten times from ten random samples of 9,000,000 unique peptides of analyzed length from the proteome of each mammalian species. In the following step, we used these 10 results for computing the mean T-distance between particular mammalian and viral proteome and its standard deviance. Standard deviation *s* was used for computing standard error (shown in the tables) and 95% confidence intervals (shown in the figures):

$$SE=\frac{s}{\sqrt{10}},$$

$$C.I.95=\frac{st_{9}\left(0.05\right)}{\sqrt{10}},$$

where *s* is the standard deviance of the T-distance and $t_{9}\left(0.05\right)$ is the critical value of Student’s t distribution with nine degrees of freedom.

To control for the richness of mammalian peptide vocabulary, we adjusted the computed distance using:

$$T_{ij}^{adj}=\frac{T_{ij}}{\frac{n-h_{i}}{n-H}},$$

where *T_ij_* is the immunological distance between *i*-th host species and *j*-th virus, *h_i_* is the size of the peptide vocabulary of a that species, *H* is the mean size of all examined host species’ peptide vocabularies, and *n* is the number of all possible peptides of a given length (20^5^ for pentapeptides and 20^6^ for hexapeptides).

SI Tables S1–S6 summarize the results for all viruses. To facilitate an easier comparison among the viruses, we applied a more complex adjustment:

$$T_{ij}^{adj}=\frac{T_{ij}}{\frac{n-h_{i}}{n-H}\cdot \frac{v_{j}}{V}},$$

where *v_j_* is the size of the peptide vocabulary of a *j*-th virus and *V* is the mean size of peptide vocabularies of all examined viruses.

### Data availability

The computer program used for building the peptide vocabularies and computation of immunological T-distances is available in a Figshare repository, [https://figshare.com/articles/software/ImunDist/17711474].

## Results

### Horseshoe bat as SARS-CoV-2 natural host

Peptide vocabularies of SARS-CoV-2 consisted of 9,175 unique tetrapeptides, 9,707 unique pentapeptides, and 9,752 unique hexapeptides. Nearly all existing 20^4^ tetrapeptides were present in the genomes of mammals. The number of tetrapeptides present in the viral peptidome but absent from the host peptidomes was thus too small (often zero) and tended to vary randomly between the mammal species. For this reason, we focused on pentapeptides and hexapeptides. We computed immunological T-distance between the pentapeptide vocabulary of the virus and corresponding vocabularies of 38 representatives of main mammalian clades as the number of different peptides in the peptidome of the virus that were absent in a random sample of 9,000,000 peptides of a given mammalian species. SI Table S1 shows immunological T-distances between the pentapeptide vocabularies of particular viruses and representatives of different clades of mammals. As expected, the smallest distance for the pentapeptides was between the SARS-CoV-2 and the great horseshoe bat *Rhinolophus ferrumequinum*. RaTG13 had the shortest distance to a representative of the Marsupialia outgroup, namely the gray short-tailed opossum *Monodelphis domestica*, but the second shortest T-distance was to the great horseshoe bat. Both viruses had also relatively short T-distances to a representative of the most basal branch of Laurasiatheria, the common shrew *Sorex araneus*. The same computation for hexapeptides showed the shortest immunological T-distance of both SARS-CoV-2 and RaTG13 to the treeshrew *Tupaia belangeri chinensis* (SI Table S2). We found no affinity of viral peptide vocabularies to the peptide vocabularies of pangolins, ferrets, dogs, or cats, i.e., several species which were originally suspected to play a role in the SARS-CoV-2 pandemic. These results agree with the currently prevailing opinion of the scientific community [6,16–20].

### Human as a natural host of the donor of spike gene

The ability of SARS CoV-2 to infect humans and spread in human populations is supposed to be related to its acquisition of a gene for the spike protein from an unknown virus of a different host specificity [4]. To test this hypothesis and to reveal the probable host specificity of the hypothetical donor of the gene for the spike protein, we repeated our analyses separately for the spike protein and the rest of the SARS-CoV-2 proteome. Results for the rest of the proteome were similar to those of the complete viral proteome but the pattern was clearer. Now, also the RaTG13 showed the shortest distance to the great horseshoe bat, while the affinity to representatives of the outgroup species decreased (SI Table S3).

**Figure 1.**
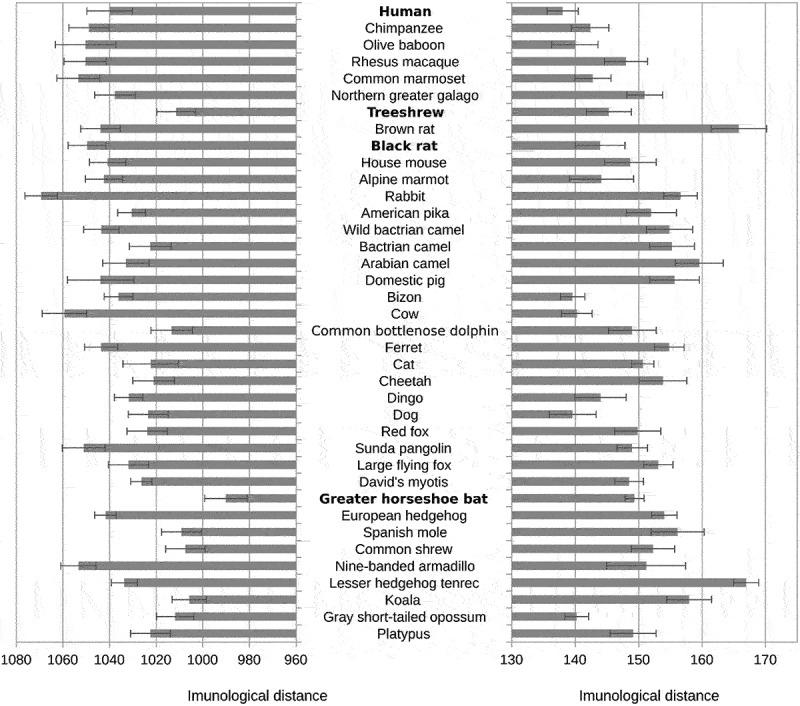
Distances between SARS-CoV-2 pentapeptide vocabulary and pentapeptide vocabularies of the main clades of mammals.

While the pentapeptide vocabulary of other genes showed the strongest affinity (shortest immunological T-distance) to the great horseshoe bat vocabulary, the pentapeptide vocabularies of the spike of both SARS-CoV-2 and RaTG13 showed the strongest affinity to the human pentapeptide vocabulary (see Figure 1 and SI Table S5).

The figure shows immunological T-distances (controlled for the size and richness of pentapeptide vocabulary of mammals) between listed mammal hosts and all proteins except spike (left) and spike protein (right) of the SARS-CoV-2 virus. The error bars show 95% confidence intervals.

### Vestiges of recent short contacts with treeshrews and rodents

**Figure 2.**
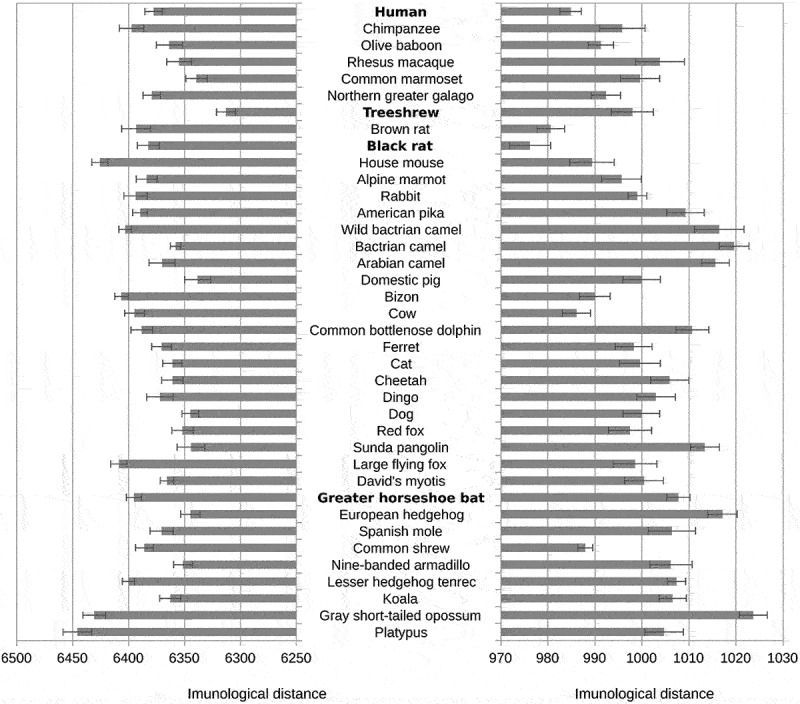
Distances between SARS-CoV-2 hexapeptide vocabulary and hexapeptide vocabularies of the main clades of mammals.

In contrast, the hexapeptide vocabulary of other genes of both SARS-CoV-2 and RaTG13 showed the shortest distance to the treeshrew, and hexapeptide vocabularies of the spike gene showed the shortest immunological T-distance to rodents: black rats in SARS-CoV-2 and mice or black rat in RaTG13 (see Figure 2 and SI Table S6).

*For the legend see*Figure 1.

The pattern observed in SARS-CoV-2 and RaTG13 is rather unique. From the set of nine other human and animal coronaviruses, only the pentapeptide vocabulary of SARS-CoV-1 showed some affinity to human pentapeptide vocabulary (SI Table S1). In SARS-CoV-1, however, the spike protein pentapeptide vocabulary showed even shorter immunological T-distance to galago and cat than to humans (SI Table S5), while the rest of the viral proteome was at a smaller pentapeptide T-distance to six unrelated species than to the great horseshoe bat (SI Table S3). The only other coronavirus that showed the strongest affinity to primate pentapeptide vocabulary (here specifically to the chimpanzee and rhesus macaque pentapeptide vocabularies) was BM48. In that case, however, it was not the spike protein but the rest of the proteome that was responsible for the observed affinity (see SI Tables S3 and S5).

## Discussion

Our results suggest that peptide vocabularies of the spike protein of SARS-CoV-2 and RaTG13 were shaped by the selection pressure of the human immune system. Their peptide vocabularies contain only a minimum of pentapeptides which are not present also in the human peptidome and can therefore serve as targets for T-cells recognition. The rest of the proteome seems to be adapted to the immune system of the horseshoe bat. This suggests that the ancestors (or a common ancestor) of SARS-CoV-2 and RaTG13, which were/was primarily adapted to the horseshoe bat, acquired the gene for the spike protein, the molecule enabling the virus to infect human cells, from another virus(es), which was/were already perfectly adapted to human hosts. Ignoring a possible but unquantifiable effect of pseudoreplication, the probability that our analyses confirm the *a priori* proposed scenario of recombination of a horseshoe bat-adapted virus with a human-adapted virus purely by chance, i.e., the probability of obtaining this result if the null hypothesis is true (p-value), can be calculated using an exact test as (1/38)(1/38). The resulting p value, 0.0007 is highly significant. Adaptation of the spike protein of SARS-CoV-2 is superior to that of RaTG13 because it additionally contains an important virulence factor, the polybasic furin cleavage site [4,10].

An unexpected result of the study was the observed affinity of hexapeptides vocabularies of SARS-CoV-2 and RaTG13 (with the exception of the spike protein) to the treeshrew vocabulary and affinity of vocabularies of the spike protein of SARS-CoV-2 and RaTG13 to that of black rats and mice, respectively. The results of a comparative study [14] showed that relative to free-living organisms, parasites have impoverished pentapeptide vocabularies and enriched hexapeptide vocabularies. It suggests that pentapeptides are the main targets of T-cells’ recognition and therefore also of the purifying selection driven by the host immune system. The length of peptides presented by MHC proteins is greater than five amino acids [21]. It is, however, known that some amino acids are needed to attach the peptide in the grove of MHC molecules and only 4–5 internal amino acids of the attached peptide probably protrude from the grove and can thus physically interact with the binding site of T-cell receptors [22].

The parasite–host similarity of hexapeptide vocabularies is therefore most likely just a side-effect of similarity of their pentapeptide vocabularies. The finding that the similarity between treeshrew hexapeptide vocabulary and viral hexapeptide vocabularies is stronger than the corresponding similarity between pentapeptide vocabularies might thus seem paradoxical. We should, however, take into account that while a short-term selection by a new host’s immune system results in a parallel impoverishing of both pentapeptide and hexapeptide vocabularies, decrease in richness of the hexapeptide vocabulary, which is a necessary side-effect of selection against pentapeptides, should proceed more quickly. Elimination of one pentapeptide from the vocabulary must automatically lead to elimination of all hexapeptides that contain this pentapeptide, which can be up to 40 different hexapeptides. The hexapeptide vocabulary of a virus could thus contain a stronger signal of recent selection by a particular host than the pentapeptide vocabulary. With this in mind, we can suggest that the small hexapeptide T-distance between both SARS-CoV-2 and RaTG13 and the treeshrew indicates that both of these viruses were recently shortly passaged in the treeshrew. This animal is relatively cheap and easily bred in captivity. Moreover, it is phylogenetically related to primates, which is why it is kept in many medical research laboratories [23]. In virological laboratories, treeshrews are often kept for the purpose of serial passage of viruses [6]. A suspiciously short immunological distance was observed also between the treeshrew and another three viruses: the SARS-CoV-1, YN2012, and BM48. It would be interesting to examine the laboratory histories of these viruses to find out whether they were passaged through the treeshrew as well.

**Figure 3.**
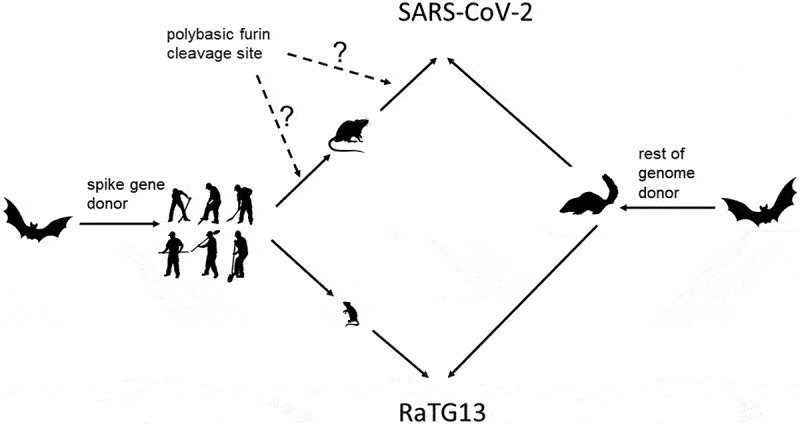
Probable origins of SARS-CoV-2 and RaTG13.

No vestiges of passaging through treeshrew were detected in the spike protein hexapeptide vocabulary. This might suggest that the progenitor(s) of SARS-CoV-2 and RaTG13 was/were passaged through the treeshrew before the current spike gene became part of its genome. In contrast, the observed similarity between the SARS-CoV-2 and RaTG13 spike protein hexapeptide vocabularies with rodent hexapeptide vocabulary might suggest that the donor(s) of the spike gene was/were passaged though rats or mice before donating the spike gene to SARS-CoV-2 and RaTG13, see Figure 3.

The “mosaic” origin of SARS-CoV-2 and RaTG13 revealed on the basis of pentapeptide and hexapeptide vocabularies. It is not possible to decide whether the plybasic furin clevage site was introduces into the S gene before or after its donor met the rodent host or even after the chimeric SARS-CoV-was formed by the recombination. The initial transmission of the donor of the spike gene from bats to humans (left) is only hypothetical and not supported by any peptide vocabularies data.

A similarity between the SARS-CoV-2 spike protein peptidome and the human peptidome has been previously reported by Kanduc and Schoenfeld [24]. They searched for evidence of a molecular mimicry between the SARS-CoV-2 spike protein and the human proteome (as a possible cause of pathogenicity of SARS-CoV-2). To this purpose, they studied hexapeptide and heptapeptide vocabularies and used the number of shared peptides (instead of the number of peptides present in the viral peptidome but missing in the host peptidome) as their criterion of affinity between viral and host genomes. The number of shared peptides does not affect the likelihood of recognition of the parasite by the host T-cells: the sole relevant criterion is the number of peptides, especially pentapeptides and tetrapeptides, which are present in the parasite’s proteins but absent in the proteins of the host. These two measures of similarity of peptide vocabularies are strongly correlated when the evolution of proteins is shaped by a genetic drift. The correlation can, however, wane or even disappear when their evolution is affected by the selection pressure of immune system of hosts.

Kanduc and Schoenfeld used a different sample of mammals: they compared three viral peptidomes with the peptidomes of four primate species (human, chimpanzee, gorilla, and macaque), and five domestic or feral species (dog, cat, rabbit, mouse, and rat). As a result, their set missed representatives of most mammal clades, including the bats and pangolins. Their results showed nearly the same similarity of SARS-CoV-2 peptidome to human and mouse peptidomes. When we repeated the analyses using Kanduc and Schoenfeld’s criterion of distance and our broader set of species, we arrived at similar results as they did, except that the hexapeptide peptidomes of viruses were more similar to mouse peptidomes than to human ones. Once we corrected the distances for peptidome size, we found that the hexapeptidome of SARS-CoV-2 was more similar to the hexapeptidome of several species, namely the baboon, marmoset, galago, dog, pangolin, bat, hedgehog, and warthog, than to the human one.

## Limitations

In the present study, we used a novel method of investigating the past host specificity of a parasite. The principle was described already in 2011 [13] and examined in detail in [14,25]. Therefore, the method has not yet been fully validated and is probably not yet optimized. For example, we do not know whether comparing of peptide vocabularies is superior to a comparison of whole peptidomes or rather of vocabularies from which very rare peptides have been removed. It is also highly probable that not only the presence of a peptide in a parasite peptidome but also its frequency plays a role in the recognition of a parasite by the immune system of a host. In the present study, we used the most conservative and simplest method of computing immunological T-distances, a method that discounts all information about the frequency of particular peptides in a peptidome. We opted for this approach to avoid the risk of data dredging. Future research may find a more accurate method of measuring immunological T-distances and studying the past and present host specificity of parasites.

## Conclusions

Both treeshrews and black rats/mice live in Central China. All of these species may well be sold in Chinese wet markets and/or be used for passaging viruses in virological laboratories. Our results therefore do not answer the question whether the new virus is the product of a natural recombination of two viruses or the outcome of deliberate insertion of the gene for the spike protein into the genome of other coronavirus species [26]. Of course, the probability of two ancestors of SARS-CoV-2 – one adapted to horseshoe bats and one to humans – being briefly passaged in two different species of laboratory animals before a natural recombination event does not seem high.
